# Optimizing Adherence to Adjuvant Imatinib in Gastrointestinal Stromal Tumor

**Published:** 2013-07-01

**Authors:** Eric D. Tetzlaff, Monica P. Davey

**Affiliations:** From Fox Chase Cancer Center, Philadelphia, Pennsylvania

## Abstract

The increasing use of patient-administered oral anticancer drugs is paralleled by new challenges in maintaining treatment adherence. These challenges are particularly significant with adjuvant therapies for prevention of disease recurrence, where the benefits of ongoing treatment are not readily apparent to patients. Nurse practitioners and physician assistants (collectively referred to as advanced practitioners) play integral roles in providing education on disease and treatment to patients that can increase adherence to oral therapies and ideally improve outcomes. For patients with gastrointestinal stromal tumor (GIST), the oral targeted therapy imatinib has become the mainstay of treatment for advanced and recurrent disease and as adjuvant therapy following surgical resection. Recent data indicate significantly improved overall survival with 3 years vs. 1 year of adjuvant imatinib therapy. Continuous dosing with imatinib is needed for optimal efficacy and to limit additional health-care costs associated with management of disease progression in GIST. However, longer duration of therapy increases the risk of nonadherence. Imatinib adherence rates, as well as factors contributing to nonadherence to adjuvant therapy in routine clinical practice, are discussed in this review. Also explored are practical approaches for improving adherence to adjuvant imatinib therapy through greater patient education, in light of the increased duration of therapy in select patients.

Gastrointestinal stromal tumor (GIST) is the most common nonepithelial tumor of the digestive tract, with an estimated incidence of 3,000 to 4,000 new cases per year in the United States (Corless & Heinrich, 2008; Nilsson et al., 2005). Gastrointestinal stromal tumors arise predominantly in the stomach (60%) and small intestine (25%); however, tumors can be found throughout the gastrointestinal tract, including the rectum, esophagus, omentum, and mesentery (Corless, Fletcher, & Heinrich, 2004; Miettinen & Lasota, 2006a). The abdominal cavity and the liver are the most common sites of distant metastasis (Miettinen & Lasota, 2006b). New diagnoses of GIST typically occur in older adults, with a median age of onset of approximately 60 years.

Surgical resection remains the standard of care for localized or potentially resectable, nonmetastatic GIST. Recurrences following surgery are common, however, occurring in > 50% of patients within 5 years. Gastrointestinal stromal tumors are refractory to conventional chemotherapy, with response rates of < 5% and a median survival < 2 years (Demetri et al., 2010). An improved understanding of the molecular underpinnings of the disease has dramatically enhanced both diagnosis and treatment. Approximately 95% of GISTs express KIT (CD117), a tyrosine kinase receptor for stem cell factor, which has become a hallmark molecular marker of this disease (Rubin et al., 2001). Gain-of-function mutations in either the KIT or platelet-derived growth factor receptor alpha (PDGFRA) gene are present in the majority of GISTs and in most cases are central to its pathogenesis (Hirota et al., 1998; 2003). These mutations have been well characterized, with most occurring in KIT exon 11 (70% of cases) or KIT exon 9 (10%–15%). Mutations in KIT and PDGFRA result in uncontrolled kinase activation that leads to increased cell proliferation and a reduction in apoptosis, contributing to oncogenic transformation.

Elucidation of the role of KIT and PDGFRA in the pathogenesis of GIST led to the use of the tyrosine kinase inhibitor imatinib (Gleevec), which is specific for these kinases, in this disease state. Treatment with imatinib has improved progression-free survival (PFS) in patients with advanced GIST, leading to its approval by the US Food and Drug Administration (FDA) for the treatment of unresectable or metastatic disease (Demetri et al., 2002; Verweij et al., 2004). Subsequently, the indication was extended to include its use as adjuvant therapy for 3 years following surgical resection of KIT-positive GIST, at a starting dose of 400 mg/day (DeMatteo et al., 2009; Novartis Pharmaceuticals, 2012). Imatinib is now recommended by the National Comprehensive Cancer Network (NCCN) treatment guidelines as first-line therapy for patients with advanced GIST and as adjuvant therapy following resection of localized tumors (NCCN, 2012a). Neoadjuvant imatinib may be an option to downstage large tumors and/or facilitate resection in some patients; this should be assessed in future clinical trials.

The optimal duration of adjuvant imatinib therapy has been the subject of debate. Data from a recently completed phase III trial demonstrated a superior survival benefit with 3 years vs. 1 year of adjuvant imatinib in patients at high risk of recurrence following surgical resection (Joensuu et al., 2012). As a direct result of this study, the FDA recently updated the product labeling to include 3 years of adjuvant therapy. Subsequently, the NCCN guidelines now recommend at least 36 months of adjuvant imatinib for patients after surgical resection of high-risk GIST, defined as tumor size > 5 cm with a mitotic rate > 5 mitoses/50 high-powered fields, or a risk of recurrence of > 50% after surgery (NCCN, 2012a; Novartis Pharmaceuticals, 2012). Although these results demonstrated an efficacy of prolonged use as adjuvant therapy of at least 3 years, the optimal duration of imatinib therapy still remains to be determined and is the subject of ongoing investigation.

It is vital for patients to adhere to the prescribed daily dosing regimen of imatinib to limit the risk of disease recurrence after primary GIST, particularly over the longer duration of therapy now recommended. Physician assistants and nurse practitioners (collectively referred to as advanced practitioners [APs]), due to their involvement in ongoing patient care, play a pivotal role in the multidisciplinary management of patients as it relates to promoting treatment adherence and persistence. This article discusses the implications and potential challenges of longer prolonged daily use of imatinib in the adjuvant setting for GIST. Specifically addressed is the need to maintain long-term patient adherence, as well as strategies the AP can use to promote adherence through adverse event management and patient education.

## Adjuvant Imatinib in GIST

Surgery remains the only potentially curative therapy and the mainstay of treatment for primary GIST. The NCCN guidelines recommend surgical resection of all GISTs > 2 cm (NCCN, 2012a). Long-term outcomes from surgery, however, are still poor due to the risk of recurrence. The overall 5-year disease-specific survival rate is 54%, with a disease-specific median survival of 66 months following resection (Demetri et al., 2010). Factors prognostic for an increased risk of recurrence include large tumor size, high mitotic index, and primary tumor located in the small bowel as well as tumor rupture (Miettinen & Lasota, 2006a).

While conventional chemotherapy and radiotherapy offer limited postsurgical benefits to patients with GIST, several studies have shown that adjuvant imatinib therapy can reduce disease recurrence. Use of adjuvant imatinib is supported by two studies conducted by the American College of Surgeons Oncology Group (ACOSOG). The first study, ACOSOG Z9000, was a single-arm, multicenter trial in which 400 mg/day imatinib was administered for 1 year to 107 patients with resected KIT+ GIST who had a high risk of recurrence (DeMatteo et al., 2009). Imatinib treatment resulted in a significantly prolonged recurrence-free survival (RFS) and a numerically improved overall survival (OS) compared with historical controls, with a median follow-up of 4 years. At 1, 2, and 3 years, RFS rates were 94%, 73%, and 61%, respectively, and OS rates were 99%, 97%, and 97%, respectively.

ACOSOG Z9001 was a pivotal phase III, double-blind, randomized trial that enrolled 713 patients who underwent resection of primary KIT+ GIST (≥ 3 cm); patients were then randomized to treatment with imatinib 400 mg daily or placebo for 1 year (DeMatteo et al., 2009). Imatinib significantly improved RFS (the primary endpoint) compared with placebo (98% vs. 83%; hazard ratio [HR], 0.35; 95% confidence interval [CI] = 0.22–0.53; * p* < .0001) at 1 year. There was not a statistically significant improvement in OS with imatinib (HR, 0.66; 95% CI = 0.22–2.03;* p* = .47); however, the trial was stopped early following interim efficacy analysis, and all patients who had received placebo were crossed over to 1 year of imatinib. This early crossover most likely confounded the long-term survival analysis of the study. An important observation from this trial was that recurrence rates increased sharply in the treatment arm after approximately 6 months of stopping imatinib. This raised the question of whether adjuvant imatinib therapy for > 1 year of duration might further reduce disease recurrence in GIST. Results of ACOSOG Z9001 led to the approval of adjuvant imatinib therapy in patients with resected KIT+ GIST.

More recently, results of the phase III Scandinavian Sarcoma Group (SSG XVIII/AIO) trial, which directly compared 1 year vs. 3 years of adjuvant therapy in patients with high-risk GIST, supported a significant benefit of prolonged imatinib therapy (Joensuu et al., 2012). In this open-label, multicenter, randomized study involving 400 patients, at a median follow-up of 54 months, the 5-year RFS was significantly longer in the 3-year arm than in the 1-year arm (65.6% vs. 47.9%; HR, 0.46 [95% CI = 0.32–0.65]; * p* < .0001). It is important to note that this trial also demonstrated significant improvement in OS with the longer duration of adjuvant imatinib (5-year OS 92.0% vs. 81.7%; HR, 0.45 [95% CI = 0.22–0.89];* p* = .019), representing the first time a clinical trial has shown an OS benefit with adjuvant therapy in GIST.

Adjuvant therapy with imatinib is rapidly being incorporated into the management of GIST in clinical practice (Bilimoria et al., 2012), and data from the SSG XVIII/AIO trial highlight the importance of maintaining adjuvant imatinib therapy for 3 years to delay disease recurrence. However, this trial has still not answered the question of the optimal duration of adjuvant imatinib therapy. The latest iteration of the NCCN guidelines recommend that "postoperative imatinib for at least 36 months should be considered for patients with high-risk GIST," based on findings of the SSG XVIII/AIO trial (Joensuu et al., 2012; NCCN, 2012a). The incidence of disease recurrence approximately 1 year after cessation of therapy in the SSG XVIII/AIO study has led investigators to question whether even longer durations may further improve outcomes. Currently, there is a clinical trial examining 5 years of adjuvant imatinib in high-risk patients (the PERSIST-5 trial, NCT00867113; ClinicalTrials.gov, 2009). With the longer-term use of imatinib therapy, sustaining patients’ adherence to therapy will become an increasing challenge for health-care providers.

## Adherence to Adjuvant Therapy: General Considerations

The term "adherence" is often considered synonymous with "persistence," but there are several key distinctions between the two that merit discussion. As defined by the International Society for Pharmacoeconomics and Outcomes Research, adherence is the extent to which patients take their medications as prescribed by their health-care provider, with respect to timing, dosage, and frequency. Persistence, on the other hand, refers to the duration between initiation and discontinuation of therapy (Cramer et al., 2008). It is vital that the AP understand that the term adherence itself carries its own implied connotations that may influence communication with patients. Adherence can suggest a more active role for the patient; there may be a perception of the patient working in concert with the health-care provider to maintain therapy, rather than the perception of "following orders" implied by the term compliance. Rates of medication adherence average 50% and can range from 0% to more than 100%, because patients may take more than the prescribed dose (Haynes, McDonald, & Garg, 2002; Osterberg & Blaschke, 2005). There is a lack of consensus as to what constitutes an adequate adherence level, with target adherence rates ranging from 80% to as high as 95% for the treatment of other serious conditions such as HIV infection (Osterberg & Blaschke, 2005).

Concerns about adherence are a relatively recent phenomenon in the oncology setting, coinciding with the introduction of oral anticancer medications. Previously, little attention was given to adherence because of the widespread use of IV chemotherapy and radiotherapy, which are typically administered in a health-care setting. However, the increased availability and use of oral anticancer agents are shifting the responsibility of administration onto the patient. As a result, oncology teams increasingly have to monitor adherence and address potential obstacles that may impact the effectiveness of treatment.

Although several methods exist for monitoring adherence (Table 1), each has its own limitations, making monitoring a difficult challenge (Osterberg & Blaschke, 2005; Ruddy, Mayer, & Partridge, 2009). Direct monitoring methods include measuring drug levels in patient serum or urine samples. Such methods may also involve the measurement of drug metabolites or biologic markers added to formulations as surrogates for ingested medicine. These approaches tend to be expensive and burdensome for both health-care providers and patients. Indirect monitoring methods can include self-reported adherence by patients or patient-completed medication diaries, pill counts, analysis of prescription refills, and use of microelectronic monitoring systems (MEMS). Maintaining accuracy with these methods is often a challenge because errors can be introduced by recall bias or entry of inaccurate information by patients. The MEMS technology may overcome some of these errors in accuracy, but it is associated with added expense.

**Table 1:**
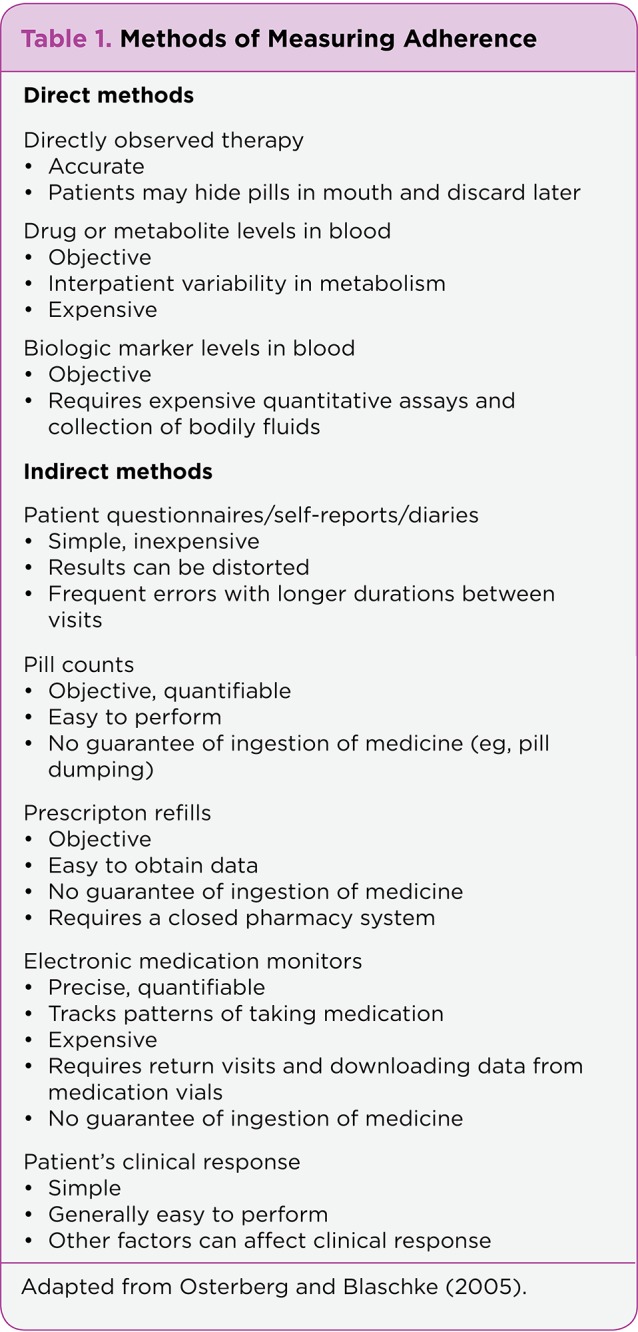
Table 1. Methods of Measuring Adherence

In general, adherence to medication is higher among patients with acute rather than chronic conditions (Osterberg & Blaschke, 2005). In both scenarios, patients experience the negative side effects of treatment, but only in the management of active disease will this be balanced with tangible treatment benefits, such as tumor shrinkage or symptom management. Maintaining patients indefinitely on preventive therapy, such as adjuvant therapy in oncology, is particularly challenging in the absence of such tangible benefits.

Patients with cancer may be presumed to be motivated to adhere to their treatment regimen due to the seriousness of their disease, yet studies of adjuvant hormonal therapy in patients with breast cancer illustrate the difficulties posed by prolonged treatment of an asymptomatic condition in which beneficial effects are only realized after a long interval. In hormone-sensitive breast cancer, adjuvant hormonal therapy with oral agents (such as tamoxifen and aromatase inhibitors) reduces disease recurrence and mortality. These agents are typically prescribed for 5 years or longer (NCCN, 2012b). Despite the known efficacy of these compounds, the rate of discontinuation with these agents is 7% to 10% per year and can be as high as 40% to 50% by 4 or 5 years (Hershman et al., 2010; Partridge et al., 2010; Partridge, Wang, Winer, & Avorn, 2003; Ruddy, Mayer, & Partridge, 2009). As a result, poor adherence to tamoxifen has been associated with shorter survival in patients with breast cancer (McCowan et al., 2008).

In addition to cancer, other examples of common, asymptomatic chronic conditions associated with suboptimal medication adherence include hypercholesterolemia and hypertension. Despite the availability of effective medications that can treat these conditions and reduce morbidity and mortality associated with cardiovascular disease, these conditions remain suboptimally managed at the population level. More than one-third of patients in the United States who are managed for hypertension have uncontrolled blood pressure (Ong, Cheung, Man, Lau, & Lam, 2007). The lack of adherence to therapy is likely a contributing factor, as studies have shown that adherence rates are similarly low (60%–75%) in patients receiving statin therapy (Jackevicius, Mamdani, & Tu, 2002).

Factors affecting adherence to adjuvant therapy can be complex, and insights may be gained from examination of lessons learned in other diseases. Several potential factors influencing adherence in the management of chronic diseases have been described (Table 2). These include patient characteristics, features associated with the treatment itself (e.g., adverse effects and increased cost), and factors related to the health-care system (Hadji, 2011; Miaskowski, Shockney, & Chlebowski, 2008; Ruddy et al., 2009). For example, in patients receiving adjuvant hormonal therapy for breast cancer, factors shown to be associated with discontinuation of therapy include treatment-related side effects, advanced age, lack of belief in the necessity and value of the medication, poor understanding of the disease itself, lack of involvement in decision-making, and poor provider support (Grunfeld, Hunter, Sikka, & Mittal, 2005; Kahn, Schneider, Malin, Adams, & Epstein, 2007; Lash, Fox, Westrup, Fink, & Silliman, 2006; Pellegrini et al., 2010).

**Table 2:**
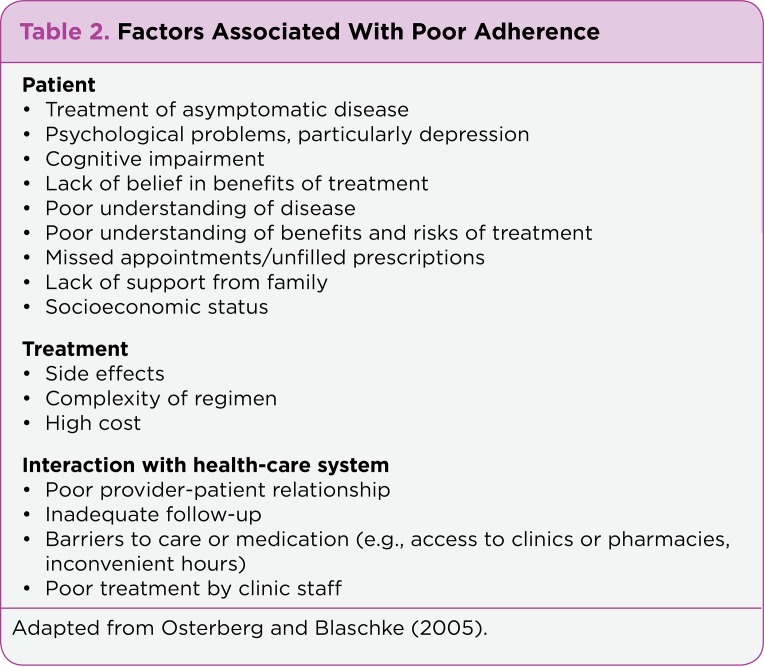
Table 2. Factors Associated With Poor Adherence

## Adherence to Imatinib Therapy

Clinical trials have established the need for continuous imatinib dosing in the management of advanced GIST (Blay et al., 2007; Le Cesne et al., 2010) and as adjuvant therapy following primary resection (Joensuu et al., 2012). Because imatinib is dosed daily, maintaining adherence is crucial in patients with GIST. However, there remains scant information on adherence to and persistence with imatinib in GIST, especially in the adjuvant setting. Recent studies have documented high rates of nonadherence to imatinib in patients with advanced GIST or chronic myelogenous leukemia (CML), as will be discussed. As noted previously, rates and factors influencing imatinib adherence may differ for advanced disease compared with the adjuvant setting.

Analysis of pharmacy claims data provides preliminary insights into the rates of adherence to imatinib therapy in routine clinical practice. In a study that tracked patient prescription data for a 24-month period in 4,043 patients with CML or GIST, compliance was 73% in patients with GIST and 78% in CML; rates of adherence declined over time in this study (Tsang, Rudychev, & Pescatore, 2006).

Another analysis of pharmacy data over a 12-month period in 320 patients with CML or GIST found that adherence assessed by a mean medication possession ratio (defined as the total day supply in the first year divided by 365) was 76% among all patients, and that > 25% of patients had interrupted imatinib therapy for at least 30 consecutive days (Feng et al., 2006). Factors identified in multivariate analyses to be associated with nonadherence to imatinib were increasing age in patients older than 51 years, female gender, increasing number of concomitant medications, and complications of disease or therapy.

Lack of adherence to imatinib therapy is a significant problem that can impact effectiveness of treatment and patient outcomes. For chronic phase CML, several recent studies in patients receiving daily imatinib assessed the impact of lack of adherence on the response to therapy. In one study, poor adherence was found to be a principal factor contributing to cytogenetic relapse and failure of therapy in patients with chronic CML who received long-term imatinib therapy (Ibrahim et al., 2011). Another study demonstrated a strong correlation between imatinib adherence and 6-year probability of a major or complete molecular response in patients with chronic-phase CML, at a median of 5 years of therapy (Marin et al., 2010).

Lastly, in the prospective Adherence Assessment with Glivec: Indicators and Outcomes (ADAGIO) study, patients with a suboptimal response had a significantly higher mean percentage of imatinib not taken (23.2%) than did those patients with an optimal response (7.3%; Noens et al., 2009). One-third of all patients in the ADAGIO study were considered nonadherent to imatinib over a 90-day period. Factors associated with lack of adherence included older age, longer time since diagnosis, longer duration of treatment, and improved health at study entry; patients also reported the "forgetfulness" factor. In patients with CML, potential relapse from nonadherence is easier to monitor via measurement of imatinib levels in blood serum; however, for patients with GIST, signs of nonadherence may not be evident until a new mass is observed via imaging.

In patients with GIST, lack of adherence to approved imatinib dosing (either physician-prescribed or patient-modulated) is fairly common in clinical practice and could contribute to patients receiving suboptimal treatment (Pisters, von Mehren, Stealey, Sirulnik, & Trent, 2010). Registry data indicate that 30% of patients with GIST received doses other than the standard 400 or 800 mg. The most common imatinib dose received was 300 mg daily in the adjuvant setting and 200 mg daily for patients with metastatic GIST. Nondaily dosing with imatinib was also frequently observed. The primary reasons for using alternative dosing regimens were toxicity and disease progression.

More information is needed on the frequency of nonadherence to imatinib, especially in the adjuvant setting, and on factors that influence adherence in these patients. Improved adherence may lead to significantly fewer hospitalizations, shorter hospital stays, and lower health-care costs overall in patients receiving imatinib (Darkow et al., 2007; Halpern, Barghoust, Mody-Patel, & Williams, 2008). For example, unadjusted medical costs were 57% to 62% lower for GIST patients with good adherence (defined as those with a ≥ 90% medication possession ratio) compared with patients with poor compliance (< 70% medication possession ratio; Halpern et al., 2008). Taken together, these data underscore the need for ensuring that patients on adjuvant treatment maintain a continuous daily administration of imatinib at standard dosing whenever possible, to provide best outcomes and optimal care.

## Management of Imatinib-Related Adverse Events in GIST

As previously discussed, the side effects that have been associated with imatinib (and other adjuvant therapies) are frequent causes of nonadherence, alternative dosing regimens, and discontinuation of therapy (Demissie, Silliman, & Lash, 2001; Feng et al., 2006; Grunfeld et al., 2005; Pisters et al., 2010). Therefore, proper management of side effects is crucial to ensure continuation of imatinib administration over prolonged periods to maximize clinical effectiveness. This is the area in which the AP can have the greatest impact through patient education.

In the Z9001 trial of adjuvant imatinib, adverse events (AEs) were the main reason for discontinuing imatinib treatment (17% of patients); these resulted in dose reduction or interruption in an additional 15% of patients (DeMatteo et al., 2009). The AEs reported in patients treated with imatinib 400 mg daily were mild to moderate in severity, the most frequent being fluid retention, gastrointestinal symptoms, fatigue, rash, and joint and muscle pain (Joensuu, Trent, & Reichardt, 2011; Novartis Pharmaceuticals, 2012). The most common AEs reported with adjuvant imatinib compared with placebo in the Z9001 trial, which are similar to those reported in trials of imatinib in the advanced GIST setting, were diarrhea, fatigue, nausea, edema, low hemoglobin, rash, vomiting, and abdominal pain; see Table 3 (Novartis Pharmaceuticals, 2012).

**Table 3:**
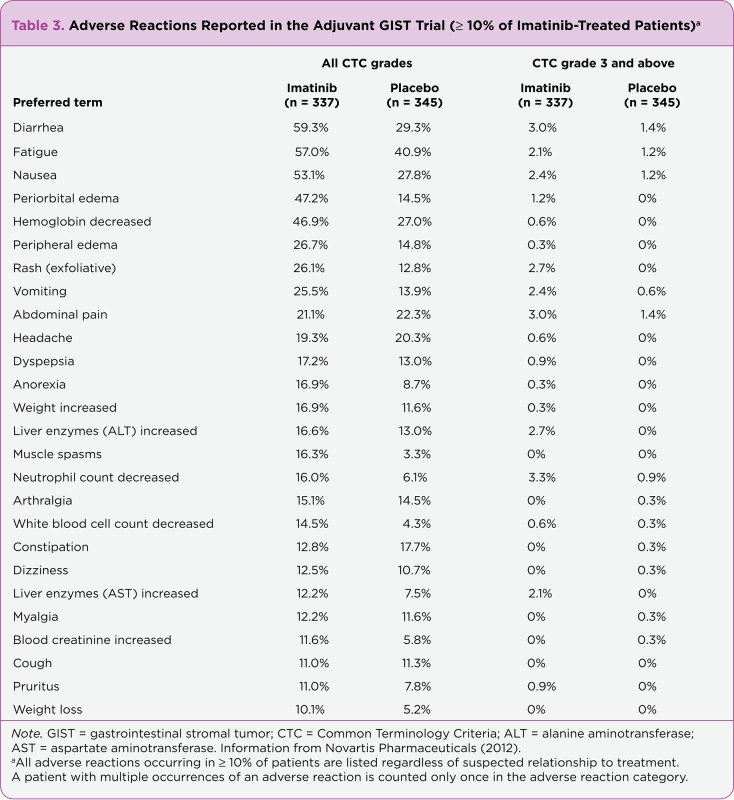
Table 3. Adverse Reactions Reported in the Adjuvant GIST Trial (≥ 10% of Imatinib-Treated Patients)

Longer administration of adjuvant imatinib therapy (i.e., 3 years) resulted in a similar AE profile compared with a shorter duration of treatment, although with an increased incidence of grade 3/4 AEs: 32.8% vs. 20.1%, respectively (Joensuu et al., 2012). There was a 14% discontinuation rate due to AEs with 3 years of imatinib compared to 8% with 1 year of adjuvant therapy; most patients seemed to experience AEs early in the treatment course, which resolved over time (Joensuu et al., 2012; Novartis Pharmaceuticals, 2012). Greater awareness of imatinib-related AEs, coupled with awareness and implementation of approaches for preventing and managing these effects, may help reduce toxicities and maximize adherence to enable more patients to maintain the recommended scheduled dosing. Table 4 summarizes established management strategies for some of the common AEs associated with imatinib therapy.

**Table 4:**
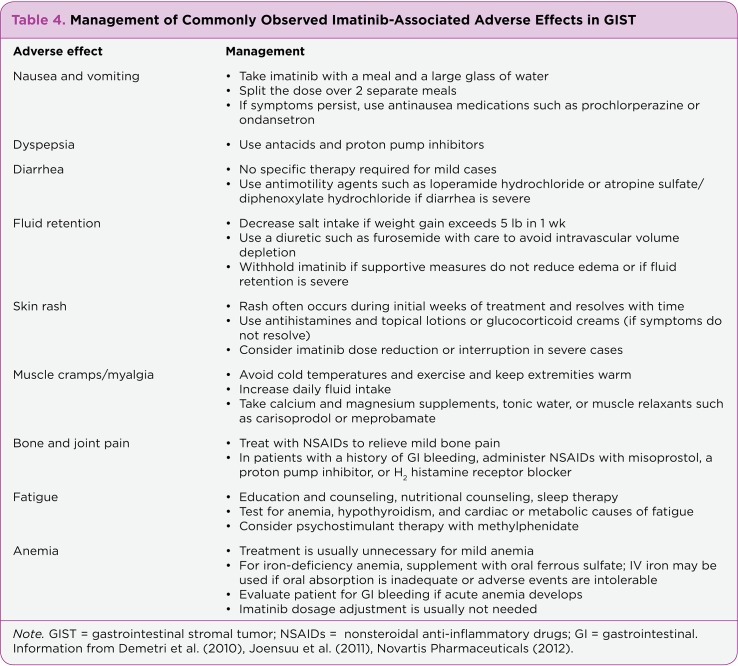
Table 4. Management of Commonly Observed Imatinib-Associated Adverse Effects in GIST

## Potential Drug Interactions

Polypharmacy is common in older patients, especially those receiving cancer therapy. Coadministration of medications can lead to drug-drug interactions, increase the risk of adverse reactions, and lower adherence. Therefore, all concomitant medications, including over-the-counter drugs and herbal remedies, should be reviewed with the patient on a regular basis to determine any potential interactions with concurrent imatinib.

Because imatinib is metabolized primarily by cytochrome P450 (CYP) 3A4 and CYP3A5 isozymes in the liver (Peng, Lloyd, & Schran, 2005), drugs that interact with these enzymes can affect imatinib pharmacokinetics (Demetri et al., 2010; Haouala et al., 2011; Novartis Pharmaceuticals, 2012). Compounds that are CYP3A4 inhibitors can decrease the metabolism of imatinib and lead to an increase in imatinib plasma concentrations. Conversely, inducers of CYP3A4 and CYP3A5 may increase imatinib metabolism, reducing a patient’s exposure to the drug. Imatinib can also influence the metabolism of drugs that are substrates of CYP3A4, potentially leading to alterations in plasma concentrations of these medications. Therefore, caution must be exercised when coadministering imatinib with CYP3A4 substrates, particularly with agents that have a narrow therapeutic window. For a list of drugs that potentially interact with imatinib and recommended management steps to take during coadministration, see Table 5. In addition, patients who have undergone large gastric resections may have absorption changes that can affect drug metabolism as well.

**Table 5:**
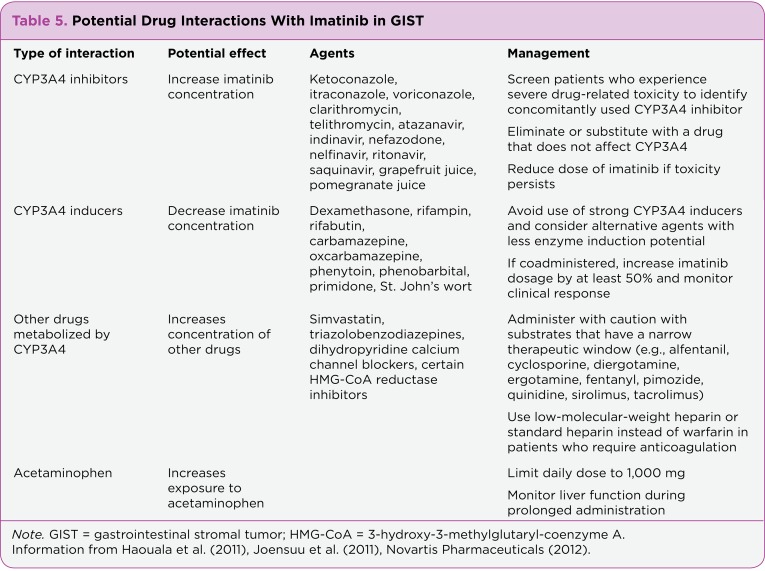
Table 5. Potential Drug Interactions With Imatinib in GIST

Protracted administration of adjuvant imatinib in patients with GIST must include a plan for regular patient monitoring. Liver function should be assessed before treatment initiation, monthly for the first 3 months, and then periodically thereafter. Regular checkups should be spaced monthly over the initial 4 to 6 months of therapy, then extended to every 3 to 6 months for 5 years, and then annually for the remainder of treatment (Griffin, Amand, & Demetri, 2005); CT scans should be done every 3 to 6 months for 5 years and then annually as well. Routine monitoring should include bloodwork as well as a physical examination. Specifically, complete blood cell count measurements should be performed weekly for the first month of treatment, then biweekly for the second month, and periodically thereafter (Novartis Pharmaceuticals, 2012). During their checkups, patients should be questioned about any ongoing or new concurrent medications. They should also be asked about any serious AEs and encouraged to seek appropriate medical care should a serious AE arise.

## Promoting Adherence to Adjuvant Imatinib

An unintended consequence of the increasing number of available oral cancer therapies has been the ongoing shift in responsibilities of treatment away from the health-care provider to the patient with cancer. Health-care providers-—including APs and nurse practitioners—will have to assume an increasingly active role in providing education to patients about their disease and treatment on a variety of fronts to maximize adherence to therapy (Barnes & Reinke, 2011; Griffin et al., 2005; Hollywood & Semple, 2001; Winkeljohn, 2007).

Because imatinib is a self-administered oral medication, patients starting imatinib need to receive detailed instructions on its correct dose and administration schedule. A primary focus must be to ensure that patients understand the disease and goals of prescribed therapy, including the benefits of maintaining continuous long-term treatment, the need to adhere to the daily treatment regimen, and the importance of refraining from discontinuing or adjusting the dose on their own. Education regarding potential side effects and their management is important to instruct patients on how to respond to toxicities and to motivate them to maintain their therapy. Patients should be taught to recognize any drug-related side effects and instructed to report them promptly to their health-care provider. Ongoing communication with APs should be emphasized. Because forgetfulness can also be a contributing factor to nonadherence, side-effect management should be emphasized before the initiation of therapy and reminded periodically. Patients should be given written and verbal instructions on which side effects or problems should be reported as well as the contact information regarding exactly when and whom to call (Winkeljohn, 2007).

Outside of regularly occurring office appointments, there are other opportunities for improving education and adherence. A patient diary that keeps track of pills, the time taken, and any symptoms can be an effective tool to promote adherence and safe administration (Hollywood & Semple, 2001). Nurse practitioners or physician assistants should spend time reviewing patient diaries at each visit. Follow-up calls to monitor adherence, evaluate early side effects, and manage symptoms provide valuable opportunities to reinforce education (Hollywood & Semple, 2001). Additionally, patients can be referred to organizations and websites that can provide information and a measure of support between visits. Patients and caregivers need to be cautioned that these groups provide a wealth of knowledge, but they are not meant to replace communication with the health-care provider.

An ongoing, supportive relationship between a patient and an AP can help to significantly bolster adherence to imatinib therapy and sustain its effectiveness through education, monitoring adherence, and managing side effects. This may be especially helpful for patients lacking family or other caregiver support. For all patients, consistent follow-up by email, phone calls, and/or contact through a physician assistant or nurse practitioner, as well as help from family members, is important for maintaining communication and providing emotional support to ensure optimal adherence.

It is important to keep in mind that patients with different economic situations may need varying levels of information or support. The financial impact of expensive therapies can influence a patient’s decision about continuing with treatment (Kelley & Venook, 2010). Patients taking oral cancer drugs may be more likely to ration or discontinue treatment than those receiving IV therapy. Advanced practitioners can help patients who have financial or reimbursement obstacles by referring them to social workers and financial counselors and providing information about the drug manufacturer’s patient assistance programs.

## Summary

The clinical benefit of prolonged adjuvant treatment with imatinib after resection of localized tumor is clear from the recent results of the SSG XVIII/AIO trial. Based on these data, current treatment guidelines recommend adjuvant imatinib for at least 3 years in patients with GIST who are at significant risk of recurrence. Despite the proven benefits of prolonged adjuvant imatinib, long-term therapy can be a challenge for some patients, leading to increased concerns related to suboptimal adherence. This is especially true for adjuvant oral cancer therapies, where long-term benefits may not be immediately apparent, but drug-related side effects are.

Reasons for treatment nonadherence vary but typically include the occurrence of treatment-related side effects and a lack of confidence in the prescribed medication. Advanced practitioners are in a prime position to improve patient awareness of disease and treatment. With adjuvant imatinib, education should involve conveying the importance of staying on therapy for a minimum of 3 years and being adherent to a continuous treatment regimen to reduce the chance of relapse. Steps should also be taken to improve expectations and management of treatment-relatedside effects. This may include increased patient education on potential side effects, more side-effect monitoring, and better, more frequent communication between patients and advanced practice clinicians. Further research on the specific reasons for imatinib nonadherence and nonstandard dosing in GIST is needed, which will ultimately advance strategies to improve adherence and patient outcomes.

## Acknowledgments

The authors take full responsibility for the content of the article but thank Gordon Strachan, PhD, of Evidence Scientific Solutions, Philadelphia, for medical editorial support funded by Novartis Pharmaceuticals
